# Parameters That May Be Used for Predicting Failure during Endoscopic Retrograde Cholangiopancreatography

**DOI:** 10.1155/2013/201681

**Published:** 2013-06-03

**Authors:** Emre Balik, Tunc Eren, Metin Keskin, Sedat Ziyade, Turker Bulut, Yilmaz Buyukuncu, Sumer Yamaner

**Affiliations:** ^1^Department of General Surgery, Istanbul Faculty of Medicine, Istanbul University, Millet Caddesi, Sehremini, Capa, Fatih, 34093 Istanbul, Turkey; ^2^Department of General Surgery, Istanbul Medeniyet University Goztepe Training and Research Hospital, Istanbul, Turkey; ^3^Department of Thoracic Surgery, Istanbul Bezmialem Vakif University, Istanbul, Turkey

## Abstract

*Aim*. Endoscopic retrograde cholangiopancreatography (ERCP) is frequently used for the diagnosis and treatment of hepatic, biliary tract, and pancreatic disorders. However, failure during cannulation necessitates other interventions. The aim of this study was to establish parameters that can be used to predict failure during ERCP. *Methods*. A total of 5884 ERCP procedures performed on 5079 patients, between 1991 and 2006, were retrospectively evaluated. *Results*. Cannulation was possible in 4482 (88.2%) patients. For each one-year increase in age, the cannulation failure rate increased by 1.01-fold (*P* = 0.002). A history of previous hepatic biliary tract surgery caused the cannulation failure rate to decrease by 0.487-fold (*P* < 0.001). A tumor infiltrating the ampulla, the presence of pathology obstructing the gastrointestinal passage, and peptic ulcer increased the failure rate by 78-, 28-, and 3.47-fold, respectively (*P* < 0.001). *Conclusions*.Patient gender and duodenal diverticula do not influence the success of cannulation during ERCP. Billroth II and Roux-en-Y gastrojejunostomy surgeries, a benign or malignant obstruction of the gastrointestinal system, and duodenal ulcers decrease the cannulation success rate, whereas a history of previous hepatic biliary tract surgery increases it. Although all endoscopists had equal levels of experience, statistically significant differences were detected among them.

## 1. Introduction

ERCP is a frequently used procedure for the diagnosis of biliary tract and pancreatic disorders. Following the first endoscopic cannulation of the ampulla of Vater by McCune, increasing experience and the technological developments in the field have enabled diagnostic and therapeutic uses of the procedure via interventions, such as sphincterotomy, biopsy of the biliary tract, extraction of calculi from the biliary tract, and stent placement, to provide temporary or permanent cures for biliary and pancreatic disorders [1–7]. Side-viewing endoscopes, supportive equipment, and improvements in visualization have helped to establish the current ERCP standards. However, difficulties imposed by the anatomy of the biliary tract and pancreas as well as the need for both an endoscopist and an endoscopy nurse with certain degrees of experience have made ERCP the most complicated, the most difficult to learn, the most interventional, and the most therapeutic of all endoscopic procedures [8]. Although the complication rates of ERCP are higher than those of other endoscopy procedures, they are markedly low compared to surgical interventions performed on the biliary tract and pancreas. The morbidity rate following ERCP is 4–15.9% (pancreatitis, 1.3–15.9%; perforation, 0.08–1.1%; bleeding, 0.76–2.3%; cholangitis, 0.57–5.01%; cholecystitis, 0.11–0.68%), whereas the mortality rate is between 0 and 1% [9, 10].

 The success of ERCP involves the cannulation of the biliary tract and obtaining a cholangiogram because cannulation is the first step for both diagnostic and therapeutic interventions [11]. Failure during cannulation renders the ERCP unsuccessful and gives rise to various consequences, including cholangitis and pancreatitis, which may require interventions, such as percutaneous transhepatic cholangiography (PTC) and surgery, with higher morbidities [12]. 

## 2. Methods

Our study involved the retrospective evaluation of 5884 ERCP procedures performed on 5079 patients by 4 experienced endoscopists at the Surgical Endoscopy Unit in the Department of General Surgery, Istanbul University Faculty of Medicine, between 1991 and 2006. The aim of the study was to establish the factors that could be used to predict cannulation failures. The cases were evaluated (with the help of a computer) with regard to the following:

1. age,
2. gender,
3. the presence of periampullary diverticula,
4. previous upper abdominal surgery,
5. the success of the cannulation,
6. the final diagnosis,
7. the endoscopist,
8. any additional findings obtained during endoscopy.

The main criterion for the success of ERCP was the cannulation of the biliary tract. The data were statistically evaluated via single- and multiple-variable analyses using SPSS 13.0 to establish factors that could be used for predicting failure during ERCP. 

## 3. Results

A total of 5884 ERCP procedures were performed on 5079 patients at the Surgical Endoscopy Unit in the Department of General Surgery, Istanbul University Faculty of Medicine, between 1991 and 2006. The procedure was performed two or more times on 688 patients. Of these 688, 197 were cases where the first intervention had failed. The age of the patients ranged between 8 and 98 years, and the mean age was estimated to be 56.2 years. The reasons for performing the ERCP procedure were as follows: jaundice in 2454 (48.3%), abdominal pain in 1906 (37.5%), cholangitis in 304 (6%), biliary fistula in 268 (5.3%), followup in 108 (2.1%) patients, and rare reasons in 39 (0.8%) patients. The rare reasons included elevated hepatic enzyme levels, pruritus, cholestatic enzyme elevation, melena, vomiting, and pancreatic fistula. 

Cannulation, which is considered to be an indicator of a successful procedure, was possible in 4482 (88.2%) patients, while the papilla could not be cannulated in 597 (11.8%) patients. A diverticulum was detected in 660 (13%) patients. A precut incision was performed in 1017 (20%) patients. A total of 873 (17.2%) patients previously had upper abdominal surgery. *Endoscopist 1* performed 2000 (39.4%), *endoscopist 2* performed 1084 (21.3%), *endoscopist 3* performed 1052 (20.7%), and *endoscopist 4* performed 943 (18.6%) of the ERCP procedures. 

The ECRP results were classified into 11 groups and are shown in detail in Table 1. 

Of the 4482 ERCP procedures where cannulation was possible, a papillotomy was performed in 3791 (84.5%). While 1377 of the procedures involved the extraction of calculi from the biliary tract, a stent was placed in the biliary tract in 507. Of the 597 patients for whom ERCP had failed, the procedure was repeated for 197 (32%) of them. A second ERCP was performed in 138 (70%) of these 197 patients. 

The data that were collected with regard to age, gender, the presence of periampullary diverticula, previous upper abdominal surgery, biliary tract cannulation, the endoscopist, and any additional findings obtained during endoscopy were evaluated with regard to their influence on the cannulation success rate using single- and multiple-variable analyses. 

The mean age for the cases where a successful cannulation could not be performed was 60.37 (±14,15). In the patients for whom a successful cannulation was conducted, the mean age was 56.66 (±16,248). This difference was statistically significant (*P* < 0.001).

When the success of the cannulation was evaluated with regard to gender, ERCP was found to have been successful in 1919 (86.8%) of the 2212 male patients and 2563 (89.4%) of the 2867 female patients. The cannulation success rate was significantly higher in women than in men (Fisher's exact test, *P* = 0.004). 

When the influence of duodenal diverticula on the cannulation success rate was evaluated, it was found that cannulation was successful in 592 (89.7%) of the 666 cases where a diverticulum was present. In contrast, cannulation was successful in 3890 (88%) of the 4419 cases where a diverticulum was not present. A statistical analysis showed that the presence of duodenal diverticula does not influence the cannulation success rate (Fisher's exact test, *P* = 0.215). 

 Of the 873 patients with a history of upper abdominal surgery, the ampulla was cannulated in 822 (94.2%) cases. Of the 4206 cases with no such history, the ampulla was cannulated in 3660 (87%) patients. The cannulation success rate in patients with a history of upper abdominal surgery was significantly higher (Fisher's exact test, *P* < 0.001). 

Previous surgical interventions were examined in detail using 6 different subgroups consisting of Billroth II gastric resection or Roux-en-Y gastrojejunostomy, hepatic resection, hydatid cyst surgery, cholecystectomy, biliary tract interventions, and so forth. A statistical analysis of these subgroups showed that the cannulation success rates in patients with Billroth II gastric resection or Roux-en-Y gastrojejunostomy were significantly lower, whereas they were significantly higher in the other groups (*P* < 0.001; Table 2). When the success rates of the four endoscopists were evaluated, *endoscopist 2* was found to be more significantly successful than the other endoscopists (*P* < 0.001; Table 3). Because each endoscopist had performed at least 943 endoscopy procedures, all of the endoscopists were considered experienced, and no comparisons were made with regard to experience. The cannulation was successful in the second ERCP attempt in 70% of the 197 patients for whom the first ERCP had failed. When the success rates of the endoscopists performing the second ERCP were evaluated, it was found that *endoscopist 4*, who had the lowest cannulation success in the first ERCP, had achieved the highest cannulation rate in the repeated ERCP procedure in the patients for whom cannulation could not be performed in the first ERCP attempt. However, this difference was not statistically significant (*P* = 0.428; Table 4). 

The distribution of the cannulation success rates with regard to the additional findings was as follows: 9.5% (*n* = 7) in 74 periampullary tumors infiltrating or distorting the ampulla, 23.6% (*n* = 13) in 55 obstructive disorders preventing gastrointestinal passage (obstructive antrum tumor, pyloric stenosis, etc.), and 84% (*n* = 27) in 38 peptic ulcer patients. Cannulation was performed in 90% (*n* = 4387) of the 4855 patients in whom no additional findings were detected. In the patients with the additional aforementioned findings, the cannulation success rate was significantly lower (chi-square test, *P* < 0.001). 

The factors that were found to produce significant differences in the single-variable analysis (i.e., age, gender, a history of upper abdominal surgery, any additional findings obtained in endoscopy, and the endoscopist) were reevaluated in a multiple-variable analysis. Multiple-variable logistic regression analysis was performed using the backward LR method. While the female gender was found to be advantageous for cannulation success rates in the single-variable analysis, the multiple-variable analysis did not reveal a statistically significant difference with regard to gender (*P* = 0.386). It was found that the cannulation failure rate increased by 1.01-fold for every one-year increase in age (*P* = 0.002). In addition, a history of previous hepatic biliary tract surgery caused the cannulation failure rate to decrease by 0.487-fold (*P* < 0.001). A tumor infiltrating the ampulla, the presence of a pathology obstructing the gastrointestinal passage, and peptic ulcer increased the failure rate by 78-, 28-, and 3.47-fold, respectively, (*P* < 0.001). In the single-variable analysis, *endoscopist 2* was the most successful. In the multiple-variable analysis, the most successful endoscopist was *endoscopist 3*. 

Accordingly, having *endoscopist 1* instead of *endoscopist 3* perform the ERCP increased the failure rate by 0.684-fold (*P* = 0.004), whereas having *endoscopist 2* perform the procedure decreased the failure rate by 0.55-fold (*P* < 0.001). No difference between *endoscopist 1* and *endoscopist 4*, who appeared to be the least successful in the single-variable analysis, was detected in the multiple-variable analysis (*P* = 0.386). The confidence intervals and relative risks in the multiple-variable analyses are shown in Table 5. 

## 4. Discussion

Success during ERCP implies the cannulation of the biliary tract and obtaining the cholangiogram because cannulation is the first step for both diagnostic and, if necessary, therapeutic interventions [11]. It should also be noted that cannulation failure renders ERCP unsuccessful and may lead to serious consequences. These include cholangitis and pancreatitis and may necessitate interventions with higher morbidities, such as PTC and surgery [12]. 

 There are few studies in the medical literature regarding age and cannulation success rates during ERCP. Lobo et al. [13] indicated that the frequency of periampullary diverticula increases significantly in patients over 75 years of age, and they found that cannulation success rates decrease significantly due to diverticula that increase with age. When evaluating our data, increasing age was found to be a risk factor for successful cannulation in the single-variable analysis. In the multiple-variable analysis, the failure rate was found to have increased by 1.01-fold for each one-year increase in the patient's age. 

There is also no data regarding the impact of gender on the cannulation success rate. In a Japanese study by Fukatsu et al., the success of ERCP was reported to be lower in women [11]. Although the cannulation success rate was found to be significantly lower in the single-variable analysis in our series, gender was not found to be a factor influencing the failure of ERCP in the multiple-variable analysis. 

 The relationship between duodenal diverticula and the cannulation success rate has been investigated in detail. There are different views regarding the effect of duodenal diverticula on cannulation. Lobo et al. [13] determined that the frequency of duodenal diverticula increases with age and decreases the cannulation success rate. They found that the success of treating intradiverticular papillas was significantly lower than that of juxtadiverticular papillas. In a study conducted on 400 patients, Boix et al. [14] detected periampullary diverticula in 131 (32.8%) patients. They classified these diverticula according to the location of the papilla: Type 1 refers to the group where the papilla is inside the diverticulum, Type 2 implies that the papilla is on the border of the diverticulum, and Type 3 means that the diverticulum is close to the papilla. Boix et al. reported that periampullary diverticula do not adversely affect cannulation. However, they concluded that cannulation is more difficult in Type 1 diverticula, and hemorrhagic complications following a sphincterotomy increase in periampullary diverticula. Fukatsu et al. [11] found a 15% frequency of duodenal diverticula in their series, and they did not consider this to be a factor influencing the cannulation success rate. In our series, duodenal diverticula were detected in 666 (13.1%) of the 5079 patients. A single-variable analysis suggested that the presence of duodenal diverticula does not influence the cannulation success rate. However, the diverticula were not classified in our study. 

 Adhesions due to previous upper abdominal surgery, gastrointestinal diversions, and gastrointestinal obstructions are also factors that affect the cannulation of the papilla during ERCP. In the series by Choudari et al. [15], Billroth I or II interventions, Roux-en-Y gastrojejunostomy, gastric outlet obstruction, and narrowing of the duodenum have been listed as reasons for ERCP failure. In a study by Baron et al. [16], Billroth II surgery, gastrojejunostomy, hepaticojejunostomy, Whipple surgery, and gastrointestinal obstructions or narrowing were reported to cause ERCP failure. In another study by Nordback and Airo [17], the ERCP success rate following gastric diversion was reported to be as low as 33%, and an inability to reach the duodenum was stated to be the most important reason. The worst results were detected in patients undergoing Billroth II surgery with a long jejunal loop. In addition, there are sources that indicate that the risk of perforation is high in patients with gastric diversion surgery since more endoscopic maneuverings are required [18]. In the study by Fukatsu et al. [11], a history of Billroth I surgery and left-lobe hypertrophy following a right hepatectomy were listed as factors influencing failure. Freemann and Guda [19] also indicated that Billroth II surgery and surgical obesity treatment increase the cannulation failure rate during ERCP. In our series, the ERCP success rate decreased to 37.5% in patients with Billroth II surgery and Roux-en-Y gastrojejunostomy, which is comparable to the findings reported in the literature. However, except for Billroth II and Roux-en-Y gastrojejunostomy interventions, the cannulation success rate was significantly higher in patients with a history of hepatic biliary tract surgery than in those with no surgical history. This group consisted of patients in whom postoperative complications (such as icterus and biliary fistula) had developed. Our interpretation of this finding is that ERCP is more successful in patients with such complications due to better endoscopist motivation or facilitated ERCP (as a result of a fixated stomach or duodenum, due to the presence of adhesions). Additionally, in the cases of malignant or benign pathologies that narrow or obstruct the gastrointestinal system, the cannulation success rate was low (23.6%) in our series, which was also comparable to the literature described above. In the patients in whom a duodenal ulcer was detected, the cannulation success rate was significantly lower than in those who did not have duodenal ulcer. In summary, while the cannulation failure rate decreased by 0.487-fold in patients with a history of hepatic biliary tract surgery, it increased by 28-fold in those with Billroth II surgery, Roux-en-Y gastrojejunostomy, and (benign or malignant) narrowing or obstruction of the gastrointestinal system. Nevertheless, it increased by 3.457-fold in those with a duodenal ulcer. 

 We detected that the cannulation failure rate increased by 78-fold in patients where periampullary tumors infiltrated or distorted the ampulla of Vater compared to patients with no such pathology. In their published series, Fukatsu et al. [11] and Freemann and Guda [19] showed that malignant biliary tract obstructions decrease the cannulation success rate during ERCP. 

 In addition to the factors that contribute to failure, factors that increase the success of ERCP have also been investigated. Of these, most data are available regarding precut (or needle-knife) incisions. In many studies, it has been reported that, in cases where cannulation cannot be performed during ERCP, a precut incision increases the success rate [11, 16, 19, 20]. Some studies have even suggested that directly starting the procedure with a precut incision, without attempting cannulation with the standard technique, is safer and more efficient than in patients where this approach is not used [17]. Because precut incisions were not directly used in our series, it was not taken into consideration in this study. In addition to these factors, it has been reported that glucagon, cholecystokinin (or its analogues), and topical nitroglycerin can be used to increase the cannulation success rate; however, it has been found that they do not produce statistically significant increases [21–23]. Such pharmacological agents were not used on the patients in our series. 

 As endoscopic sonography has become available, it has begun to be used in ERCP procedures where cannulation cannot be performed. In a study by Gupta et al. [24], it was found that in the ERCP procedures where cannulation cannot be performed using the standard technique, endosonography decreases the need for drainage via PTC or surgical procedures. It was also emphasized that endosonography can be used to facilitate cannulation during ERCP. Endosonography is not used in our clinic. 

 Another factor that influences the ERCP success rate is the experience of the endoscopist. The success rate increases in direct proportion to experience. Although various sources have reported different figures, it has been suggested that, during his or her training, an endoscopist should have performed approximately 100–200 ERCPs with a successful cannulation rate of 85–90%, and at least 25 (preferably half) of these interventions should have been therapeutic procedures [25, 26]. In an American study by Verma et al. [27], the cannulation success rate increased from 43% to 80% after 350 ERCPs, whereas it was found to be more than 96% after 400 ERCPs. In our series, all of the endoscopists had experience with at least 3000 gastroscopy and colonoscopy procedures. The fewest number of ERCP procedures that an endoscopist had performed was 943. Therefore, all four endoscopists were considered to be experienced. However, the single-variable analysis showed that the highest cannulation rate was achieved by *endoscopist 2*, and this difference was statistically significant. However, in the cases where a second ERCP was performed after an unsuccessful first ERCP attempt, *endoscopist 4*, who seemed to be the least successful endoscopist in the single-variable analysis, was found to be the most successful, although this difference was not statistically significant. Despite the fact that all of the endoscopists were equally experienced, the multiple-variable analysis showed that the cannulation success rates were significantly higher for *endoscopist 2* and *endoscopist 3*. 

Ramirez et al. [28] showed that when the same individual performs a second ERCP after a first failed attempt, the success rate increases from 87.5% to 95%. Our series showed that for failed ERCPs, the success rate was more than 95% when we performed a second ERCP. 

## 5. Conclusions

 We conclude that the patient gender and duodenal diverticula do not influence the cannulation success rate during ERCP. In contrast, Billroth II and Roux-en-Y gastrostomy surgeries, a benign or malignant gastrointestinal obstruction that prevents the passage of the endoscope, and duodenal ulcers decrease the cannulation success rate, whereas a history of previous hepatic biliary tract surgery increases the success rate. In addition, although all of the endoscopists had equal levels of experience, statistically significant differences were detected among them. 

## Figures and Tables

**Table 1:** ERCP diagnosis.

Final diagnosis	*n*	%
Biliary tract stones	952	18.7
Malignant obstruction of the biliary tract	778	15.4
Normal ERCP	661	13
Unsuccessful ERCP	597	11.7
Stones in the gall bladder and in the biliary tract	593	11.6
Gall bladder stones	515	10.2
Enlarged biliary tract	508	10.1
Biliary fistula	187	3.7
Benign obstruction of the biliary tract	139	2.7
Cystic disease of the biliary tract	29	0.6
Others	120	2.3

Total	5079	100

**Table 2:** Distribution of cannulation success rates for each previous upper abdominal surgery intervention subgroup (chi-square test, *P* < 0.001).

Surgical history	Cannulation		Total
	Yes	No	
None	552 (13.1%)	3665 (86.9%)	4217 (100%)
Billroth II/R-Y gastrojejunostomy	10 (62.5%)	6 (37.5%)	16 (100%)
Hepatic resection	1 (10%)	9 (90%)	10 (100%)
Hydatid cyst surgery	4 (6.1%)	62 (93.9%)	66 (100%)
Cholecystectomy	20 (3.2%)	614 (96.8%)	634 (100%)
Biliary tract exploration	8 (7%)	107 (93%)	115 (100%)
Others	2 (9.5%)	19 (90.5%)	21 (100%)

Total	597 (11.8%)	4482 (88.2%)	5079 (100%)

**Table 3:** Distribution of cannulation success rates for each endoscopist (chi-square test, *P* < 0.001).

Endoscopist	Cannulation		Total
	Yes	No	
*Endoscopist 1 *	262 (13.1%)	1738 (86.9%)	2000 (100%)
*Endoscopist 2 *	91 (8.4%)	993 (91.6%)	1084 (100%)
*Endoscopist 3 *	108 (10.3%)	944 (89.7%)	1052 (100%)
*Endoscopist 4 *	136 (14.4%)	807 (85.6%)	943 (100%)

Total	597 (11.2%)	4482 (88.2%)	5079 (100%)

**Table 4:** Distribution of cannulation success rates for each endoscopists in cases where a second ERCP was performed after a failed first ERCP (chi-square test, *P* = 0.428).

Endoscopist	Cannulation		Total
	No	Yes	
*Endoscopist 1 *	33 (35.5%)	60 (64.5%)	93 (100%)
*Endoscopist 2 *	9 (23.7%)	29 (76.3%)	38 (100%)
*Endoscopist 3 *	10 (27.8%)	26 (72.2%)	36 (100%)
*Endoscopist 4 *	7 (23.3%)	23 (76.7%)	30 (100%)

Total	59 (29.9%)	138 (70.1%)	197 (100%)

**Table 5:** Distribution of the predicted relative risk (PRR) and confidence intervals (CIs) for the factors influencing cannulation in the multiple-variable analysis (logistic regression using the backward LR method).

	PRR		95% confidence interval	
			Minimum	Maximum
Factors				

*P* = 0.002	Age	1.01	1.004	1.016
*P* = 0.386	Gender	1.086	0.901	1.309
*P* < 0.000	History of previous upper abdominal surgery	0.467	0.357	0.663

Additional findings				

*P* = 0.000	Periampullary tumor infiltrating the ampulla	78.060	35.426	172.001
*P* = 0.000	Problem in GIT passage	29.190	15.413	55.282
*P* = 0.001	Peptic ulcer	3.457	1.687	7.085

Endoscopist				

	*Endoscopist 1 *			
*P* = 0.000	*Endoscopist 2 *	0.550	0.419	0.722
*P* = 0.004	*Endoscopist 3 *	0.684	0.529	0.883
*P* = 0.389	*Endoscopist 4 *	0.897	0.700	1.149
